# Supplementary material for: A community-informed microplastic exposure index based on Pacific Island fisheries: Integrating local knowledge with empirical data
Source: Camb Prism Coast Futur. 2026 Apr 14;4:e8. doi: 10.1017/cft.2026.10027 (PMC13107359; doi:10.1017/cft.2026.10027)

**Supplementary Information**

**A Community-Informed Microplastic Exposure Index Based on Pacific Island Fisheries: Integrating Local Knowledge with Empirical Data**

*Authors and affiliations*

Amanda Kirsty Ford^1^*, Salanieta Kitolelei^1,2^, Rufino Varea^3^, Joycinette Vosumbe Botleng^1^, June Brian Molitaviti^4^, Semese Alefaio^5^, Lavata Nivaga^6^, Kelly T. Brown^1^, Cherie Morris^1^, Eseta Drova^1^, Brian L. Stockwell^1^, Siutiti Fe’ao^7,8^, and Jasha Dehm^1^

^1^ Centre for Sustainable Futures, The University of the South Pacific, Suva, Fiji

^2^ Leibniz Centre for Tropical Marine Research, Bremen, Germany

^3^ Office of the Secretariat, Pacific Islands Climate Action Network, Suva, Fiji

^4^ Vanuatu Fisheries Department, Government of Vanuatu

^5^ Tuvalu Fisheries Authority, Ministry of Natural Resources Development, Tuvalu

^6^ Live and Learn, Funafuti, Tuvalu

^7^ School of Agriculture, Geography, Environment, Ocean and Natural Sciences, The University of the South Pacific, Suva, Fiji

^8^ Graduate School of Bioresource and Bioenvironmental Sciences, Kyushu University, Japan

* Corresponding author: Amanda Kirsty Ford (amanda.ford@usp.ac.fj)

**Supplementary Table 1**: Population statistics of fisher interviewees who participated in this research, including the number (#) and relative percentage (%) of men and women interviewed per country.

| **Country** | **Dates** | **Total # of interviews** | **# men** | **% men** | **# women** | **% women** |
| --- | --- | --- | --- | --- | --- | --- |
| Fiji | Jul-Aug 2023 | 28 | 16 | 60 | 12 | 40 |
| Tonga | Nov 2023-Feb 2024 | 30 | 21 | 70 | 9 | 30 |
| Tuvalu | Jul-Nov 2024 | 29 | 19 | 60 | 10 | 40 |
| Vanuatu | Jun-Sep 2024 | 23 | 11 | 50 | 12 | 50 |
| Total | | 110 | 67 | 60 | 43 | 40 |

**Supplementary Table 2:** Table showing the genera and species sampled for microplastics within each family included in the Exposure Index assessments, presented by country.

| **Country** | **Family** | **Genus** | **Species** |
| --- | --- | --- | --- |
| Fiji | Hemiramphidae | *Hemiramphus* | *Hemiramphus far* |
| Fiji | Lethrinidae | *Lethrinus* | *Lethrinus amboinensis* |
| Fiji | Lethrinidae | *Lethrinus* | *Lethrinus harak* |
| Fiji | Lethrinidae | *Lethrinus* | *Lethrinus lentjan* |
| Fiji | Lethrinidae | *Lethrinus* | *Lethrinus olivaceus* |
| Fiji | Lutjanidae | *Lutjanus* | *Lutjanus fulvus* |
| Fiji | Lutjanidae | *Lutjanus* | *Lutjanus gibbus* |
| Fiji | Lutjanidae | *Lutjanus* | *Lutjanus russellii* |
| Fiji | Lutjanidae | *Lutjanus* | *Lutjanus semicinctus* |
| Fiji | Mullidae | *Parupeneus* | *Parupeneus barberinus* |
| Fiji | Scaridae | *Cetoscarus* | *Cetoscarus ocellatus* |
| Fiji | Scombridae | *Rastrelliger* | *Rastrelliger brachysoma* |
| Fiji | Serranidae | *Epinephelus* | *Epinephelus timorensis* |
| Fiji | Serranidae | *Plectropomus* | *Plectropomus leopardus* |
| Fiji | Siganidae | *Siganus* | *Siganus vermiculatus* |
| Fiji | Sphyraenidae | *Sphyraena* | *Sphyraena barracuda* |
| Fiji | Terapontidae | *Terapon* | *Terapon jarbua* |
| Tonga | Acanthuridae | *Acanthurus* | *Acanthurus dussumieri* |
| Tonga | Acanthuridae | *Acanthurus* | *Acanthurus mata* |
| Tonga | Acanthuridae | *Naso* | *Naso brevirostris* |
| Tonga | Acanthuridae | *Naso* | *Naso unicornis* |
| Tonga | Carangidae | *Caranx* | *Caranx melampygus* |
| Tonga | Carangidae | *Selar* | *Selar crumenophthalmus* |
| Tonga | Labridae | *Cheilinus* | *Cheilinus trilobatus* |
| Tonga | Labridae | *Oxycheilinus* | *Oxycheilinus digramma* |
| Tonga | Lethrinidae | *Lethrinus* | *Lethrinus erythracanthus* |
| Tonga | Lethrinidae | *Lethrinus* | *Lethrinus harak* |
| Tonga | Lethrinidae | *Lethrinus* | *Lethrinus miniatus* |
| Tonga | Lethrinidae | *Lethrinus* | *Lethrinus nebulosus* |
| Tonga | Lutjanidae | *Aprion* | *Aprion virescens* |
| Tonga | Lutjanidae | *Lutjanus* | *Lutjanus fulvus* |
| Tonga | Lutjanidae | *Lutjanus* | *Lutjanus gibbus* |
| Tonga | Mugillidae | *Crenimugli* | *Crenimugil crenilabis* |
| Tonga | Mugillidae | *Ellochelon* | *Ellochelon vaigiensis* |
| Tonga | Mullidae | *Parupeneus* | *Parupeneus barberinus* |
| Tonga | Mullidae | *Parupeneus* | *Parupeneus heptacanthus* |
| Tonga | Mullidae | *Parupeneus* | *Parupeneus indicus* |
| Tonga | Scaridae | *Calotomus* | *Calotomus carolinus* |
| Tonga | Scaridae | *Hipposcarus* | *Hipposcarus longiceps* |
| Tonga | Scaridae | *Scarus* | *Scarus dimidiatus* |
| Tonga | Scaridae | *Scarus* | *Scarus prasiognathos* |
| Tonga | Scaridae | *Scarus* | *Scarus rivulatus* |
| Tonga | Serranidae | *Epinephelus* | *Epinephelus areolatus* |
| Tonga | Serranidae | *Epinephelus* | *Epinephelus cyanopodus* |
| Tonga | Serranidae | *Epinephelus* | *Epinephelus maculatus* |
| Tonga | Siganidae | *Siganus* | *Siganus lineatus* |
| Tonga | Siganidae | *Siganus* | *Siganus punctatus* |
| Tuvalu | Acanthuridae | *Acanthurus* | *Acanthurus auranticavus* |
| Tuvalu | Acanthuridae | *Acanthurus* | *Acanthurus lineatus* |
| Tuvalu | Acanthuridae | *Acanthurus* | *Acanthurus mata* |
| Tuvalu | Acanthuridae | *Acanthurus* | *Acanthurus nigricauda* |
| Tuvalu | Acanthuridae | *Acanthurus* | *Acanthurus triostegus* |
| Tuvalu | Acanthuridae | *Acanthurus* | *Acanthurus xanthopterus* |
| Tuvalu | Acanthuridae | *Naso* | *Naso annulatus* |
| Tuvalu | Acanthuridae | *Naso* | *Naso lituratus* |
| Tuvalu | Acanthuridae | *Naso* | *Naso unicornis* |
| Tuvalu | Holocentridae | *Myripristis* | *Myripristis adusta* |
| Tuvalu | Holocentridae | *Myripristis* | *Myripristis berndti* |
| Tuvalu | Holocentridae | *Myripristis* | *Myripristis murdjan* |
| Tuvalu | Holocentridae | *Sargocentron* | *Sargocentron spiniferum* |
| Tuvalu | Lethrinidae | *Gnathodentex* | *Gnathodentex aureolineatus* |
| Tuvalu | Lethrinidae | *Lethrinus* | *Lethrinus erythracanthus* |
| Tuvalu | Lethrinidae | *Lethrinus* | *Lethrinus harak* |
| Tuvalu | Lethrinidae | *Lethrinus* | *Lethrinus obsoletus* |
| Tuvalu | Lethrinidae | *Lethrinus* | *Lethrinus olivaceus* |
| Tuvalu | Lethrinidae | *Lethrinus* | *Lethrinus xanthochilus* |
| Tuvalu | Lethrinidae | *Monotaxis* | *Monotaxis grandoculis* |
| Tuvalu | Lethrinidae | *Monotaxis* | *Monotaxis heterodon* |
| Tuvalu | Lutjanidae | *Aphareus* | *Aphareus rutilans* |
| Tuvalu | Lutjanidae | *Lutjanus* | *Lutjanus gibbus* |
| Tuvalu | Lutjanidae | *Lutjanus* | *Lutjanus monostigma* |
| Tuvalu | Lutjanidae | *Macolor* | *Macolor niger* |
| Tuvalu | Mullidae | *Mulloidichthys* | *Mulloidichthys flavolineatus* |
| Tuvalu | Mullidae | *Mulloidichthys* | *Mulloidichthys vanicolensis* |
| Tuvalu | Mullidae | *Parupeneus* | *Parupeneus barberinus* |
| Tuvalu | Mullidae | *Parupeneus* | *Parupeneus multifasciatus* |
| Tuvalu | Scaridae | *Hipposcarus* | *Hipposcarus longiceps* |
| Tuvalu | Scaridae | *Scarus* | *Scarus altipinnis* |
| Tuvalu | Serranidae | *Anyperodon* | *Anyperodon leucogrammicus* |
| Tuvalu | Serranidae | *Epinephelus* | *Epinephelus howlandi* |
| Tuvalu | Serranidae | *Epinephelus* | *Epinephelus maculatus* |
| Tuvalu | Serranidae | *Epinephelus* | *Epinephelus merra* |
| Tuvalu | Serranidae | *Epinephelus* | *Epinephelus polyphekadion* |
| Tuvalu | Serranidae | *Epinephelus* | *Epinephelus sp.* |
| Tuvalu | Serranidae | *Epinephelus* | *Epinephelus spilotoceps* |
| Tuvalu | Siganidae | *Siganus* | *Siganus argenteus* |
| Tuvalu | Siganidae | *Siganus* | *Siganus punctatus* |
| Tuvalu | Sphyraenidae | *Sphyraena* | *Sphyraena obtusata* |
| Tuvalu | Sphyraenidae | *Sphyraena* | *Sphyraena forsteri* |
| Vanuatu | Acanthuridae | *Acanthurus* | *Acanthurus lineatus* |
| Vanuatu | Acanthuridae | *Acanthurus* | *Acanthurus maculiceps* |
| Vanuatu | Acanthuridae | *Acanthurus* | *Acanthurus nigroris* |
| Vanuatu | Acanthuridae | *Acanthurus* | *Acanthurus olivaceus* |
| Vanuatu | Acanthuridae | *Acanthurus* | *Acanthurus triostegus* |
| Vanuatu | Acanthuridae | *Ctenochaetus* | *Ctenochaetus striatus* |
| Vanuatu | Belonidae | *Tylosurus* | *Tylosurus crocodilus* |
| Vanuatu | Carangidae | *Carangoides* | *Ferdauia ferdau* |
| Vanuatu | Carangidae | *Caranx* | *Caranx ignobilis* |
| Vanuatu | Carangidae | *Caranx* | *Caranx melampygus* |
| Vanuatu | Carangidae | *Caranx* | *Caranx papuensis* |
| Vanuatu | Carangidae | *Caranx* | *Caranx sexfasciatus* |
| Vanuatu | Carangidae | *Selar* | *Selar crumenophthalmus* |
| Vanuatu | Carangidae | *Seriola* | *Seriola dumerili* |
| Vanuatu | Carangidae | *Seriola* | *Seriola rivoliana* |
| Vanuatu | Haemulidae | *Plectorhinchus* | *Plectorhinchus lineatus* |
| Vanuatu | Haemulidae | *Plectorhinchus* | *Plectorhinchus vittatus* |
| Vanuatu | Hemiramphidae | *Hemiramphus* | *Hemiramphus far* |
| Vanuatu | Kyphosidae | *Kyphosus* | *Kyphosus cinerascens* |
| Vanuatu | Kyphosidae | *Kyphosus* | *Kyphosus vaigiensis* |
| Vanuatu | Labridae | *Cheilinus* | *Cheilinus trilobatus* |
| Vanuatu | Labridae | *Cheilio* | *Cheilio inermis* |
| Vanuatu | Labridae | *Oxycheilinus* | *Oxycheilinus digramma* |
| Vanuatu | Labridae | *Novaculichthys* | *Novaculichthys taeniourus* |
| Vanuatu | Labridae | *Cheilinus* | *Cheilinus chlorourus* |
| Vanuatu | Lethrinidae | *Gymnocranius* | *Gymnocranius grandoculis* |
| Vanuatu | Lethrinidae | *Lethrinus* | *Lethrinus atkinsoni* |
| Vanuatu | Lethrinidae | *Lethrinus* | *Lethrinus harak* |
| Vanuatu | Lethrinidae | *Lethrinus* | *Lethrinus nebulosus* |
| Vanuatu | Lethrinidae | *Lethrinus* | *Lethrinus obsoletus* |
| Vanuatu | Lethrinidae | *Lethrinus* | *Lethrinus olivaceus* |
| Vanuatu | Lethrinidae | *Monotaxis* | *Monotaxis grandoculis* |
| Vanuatu | Lutjanidae | *Aphareus* | *Aphareus rutilans* |
| Vanuatu | Lutjanidae | *Aprion* | *Aprion virescens* |
| Vanuatu | Lutjanidae | *Etelis* | *Etelis radiosus* |
| Vanuatu | Lutjanidae | *Lutjanus* | *Lutjanus argentimaculatus* |
| Vanuatu | Lutjanidae | *Lutjanus* | *Lutjanus bohar* |
| Vanuatu | Lutjanidae | *Lutjanus* | *Lutjanus fulvus* |
| Vanuatu | Lutjanidae | *Lutjanus* | *Lutjanus kasmira* |
| Vanuatu | Lutjanidae | *Paracaesio* | *Paracaesio kusakarii* |
| Vanuatu | Lutjanidae | *Pristipomoides* | *Pristipomoides argyrogrammicus* |
| Vanuatu | Lutjanidae | *Pristipomoides* | *Pristipomoides auricilla* |
| Vanuatu | Lutjanidae | *Pristipomoides* | *Pristipomoides filamentosus* |
| Vanuatu | Lutjanidae | *Pristipomoides* | *Pristipomoides flavipinnis* |
| Vanuatu | Lutjanidae | *Pristipomoides* | *Pristipomoides multidens* |
| Vanuatu | Lutjanidae | *Pristipomoides* | *Pristipomoides typus* |
| Vanuatu | Mugilidae | *Crenimugil* | *Crenimugil seheli* |
| Vanuatu | Mugilidae | *Ellochelon* | *Ellochelon vaigiensis* |
| Vanuatu | Mullidae | *Mulloidichthys* | *Mulloidichthys flavolineatus* |
| Vanuatu | Mullidae | *Mulloidichthys* | *Mulloidichthys vanicolensis* |
| Vanuatu | Mullidae | *Parupeneus* | *Parupeneus barberinus* |
| Vanuatu | Mullidae | *Parupeneus* | *Parupeneus crassilabris* |
| Vanuatu | Mullidae | *Parupeneus* | *Parupeneus cyclostomus* |
| Vanuatu | Mullidae | *Parupeneus* | *Parupeneus indicus* |
| Vanuatu | Mullidae | *Parupeneus* | *Parupeneus multifasciatus* |
| Vanuatu | Scaridae | *Calotomus* | *Calotomus carolinus* |
| Vanuatu | Scaridae | *Cetoscarus* | *Cetoscarus ocellatus* |
| Vanuatu | Scaridae | *Chlorurus* | *Chlorurus bleekeri* |
| Vanuatu | Scaridae | *Chlorurus* | *Chlorurus microrhinos* |
| Vanuatu | Scaridae | *Chlorurus* | *Chlorurus spilurus* |
| Vanuatu | Scaridae | *Scarus* | *Scarus altipinnis* |
| Vanuatu | Scaridae | *Scarus* | *Scarus chameleon* |
| Vanuatu | Scaridae | *Scarus* | *Scarus frenatus* |
| Vanuatu | Scaridae | *Scarus* | *Scarus niger* |
| Vanuatu | Scaridae | *Scarus* | *Scarus oviceps* |
| Vanuatu | Scaridae | *Scarus* | *Scarus psittacus* |
| Vanuatu | Scaridae | *Scarus* | *Scarus quoyi* |
| Vanuatu | Scaridae | *Scarus* | *Scarus rivulatus* |
| Vanuatu | Scaridae | *Scarus* | *Scarus rubroviolaceus* |
| Vanuatu | Serranidae | *Cephalopholis* | *Cephalopholis cyanostigma* |
| Vanuatu | Serranidae | *Cephalopholis* | *Cephalopholis urodeta* |
| Vanuatu | Serranidae | *Epinephelus* | *Epinephelus maculatus* |
| Vanuatu | Serranidae | *Variola* | *Variola albimarginata* |
| Vanuatu | Serranidae | *Variola* | *Variola louti* |
| Vanuatu | Siganidae | *Siganus* | *Siganus canaliculatus* |
| Vanuatu | Siganidae | *Siganus* | *Siganus corallinus* |
| Vanuatu | Siganidae | *Siganus* | *Siganus spinus* |

**Supplementary Table 3:** Table showing the list of unique taxa fishers identified within each family as being within the top 10 species caught within their fishing grounds, with the number of times they were mentioned in each country.

| **Country** | **Family** | **Taxa Identified by Fishers** | **Mentions** |
| --- | --- | --- | --- |
| Fiji | Acanthuridae | *Balagi* (Acanthuridae) | 2 |
| Fiji | Acanthuridae | *Dridri* (*Ctenoechaetus* spp.) | 3 |
| Fiji | Acanthuridae | *ika loa* (*Ctenochaetus striatus*) | 1 |
| Fiji | Acanthuridae | *Sivisivi* (*Ta*) (*Naso* spp) | 2 |
| Fiji | Acanthuridae | *Ta* (*Naso unicornis*) | 4 |
| Fiji | Balistidae | *Cumu* (Balistidae) | 4 |
| Fiji | Balistidae | *Qau* (*Pseudobalistes viridescens*) | 1 |
| Fiji | Carangidae | *Dole* (*Caranx* spp*.*) | 2 |
| Fiji | Carangidae | *Jule* (*Selar crumenophthalmus*) | 1 |
| Fiji | Carangidae | *Kaikai* (Carangidae) | 4 |
| Fiji | Carangidae | *Saqa* (*Caranx ignobillis*) | 7 |
| Fiji | Carangidae | *Saqa* (*Caranx* spp*.*) | 11 |
| Fiji | Carangidae | *Vilu* (*Gnathanodon speciosus*) | 1 |
| Fiji | Carcharhinidae | *Qio* (*Carcharhinus* spp.) | 1 |
| Fiji | Clupeidae | *Daniva* (*Herklotsichthys quadrimaculatus*) | 2 |
| Fiji | Diodontidae | *Sokisoki* (*Diodon* spp.) | 1 |
| Fiji | Eleotridae | *Kurukoto* (*Eleotris melanosoma*) | 1 |
| Fiji | Gerreidae | *Matu* (*Gerres* spp.) | 1 |
| Fiji | Haemulidae | *Drekeni* (*Plectorhinchus gibbosus*) | 3 |
| Fiji | Hemiramphidae | *Buhe* (*Hemiramphus lutkei*) | 4 |
| Fiji | Hemiramphidae | *Busa* (Hemiramphidae) | 3 |
| Fiji | Labridae | *Drevu* (Labridae) | 1 |
| Fiji | Labridae | *Tuvukeli* (Labridae) | 1 |
| Fiji | Lethrinidae | *Bu* (*Monotaxis grandoculis*) | 1 |
| Fiji | Lethrinidae | *Cabuju* (*Lethrinus* spp.) | 3 |
| Fiji | Lethrinidae | *Dokonivudi* (*Lethrinus* spp.) | 1 |
| Fiji | Lethrinidae | *Gusula* (*Lethrinus* spp.) | 1 |
| Fiji | Lethrinidae | *Kabatia* (*Lethrinus harak*) | 22 |
| Fiji | Lethrinidae | *Kacika* (Lethrinidae) | 2 |
| Fiji | Lethrinidae | *Kawaqo* (*Lethrinus* spp.) | 1 |
| Fiji | Lethrinidae | *Sabutu* (*Lethrinus* spp.) | 5 |
| Fiji | Lethrinidae | *Vuvunivudi* (Lethridnidae) | 1 |
| Fiji | Lutjanidae | *Boca* (*Lutjanus gibbus*) | 1 |
| Fiji | Lutjanidae | *Damu* (*Lutjanus* spp.) | 11 |
| Fiji | Lutjanidae | *Kake* (Lutjanidae) | 6 |
| Fiji | Lutjanidae | *Qitawa* (Lutjanidae) | 3 |
| Fiji | Lutjanidae | *Kake* (Lutjanidae) | 1 |
| Fiji | Mugilidae | *Kanace* (*Crennimugil* spp.) | 10 |
| Fiji | Mullidae | *Cucu* (*Parupeneus indicus*) | 1 |
| Fiji | Mullidae | *Donau* (Mullidae) | 1 |
| Fiji | Muraenidae | *Dabea* (*Gymnothorax* spp.) | 2 |
| Fiji | Scaridae | *Karakarawa* (Scaridae) | 6 |
| Fiji | Scaridae | *Rawarawa* (*Scarus rivulatus*) | 3 |
| Fiji | Scaridae | *Ulavi* (Scaridae) | 2 |
| Fiji | Scombridae | *Salala* (*Rastrelliger* spp.) | 5 |
| Fiji | Scombridae | Tuna (*Thunnus* spp.) | 2 |
| Fiji | Scombridae | Wahoo (*Acanthocybium solandri*) | 1 |
| Fiji | Scombridae | *Walu* (*Scomberomonis commerson*) | 6 |
| Fiji | Serranidae | *Dalobulewa* (*Cephalopholis sonnerati*) | 1 |
| Fiji | Serranidae | *Donu* (*Plectropomus* spp.) | 5 |
| Fiji | Serranidae | *Kasala* (*Epinephelus* spp.) | 1 |
| Fiji | Serranidae | *Kawakawa* (*Epinephelus* spp.) | 14 |
| Fiji | Serranidae | *Tokula* (*Epinephelus* spp.) | 4 |
| Fiji | Serranidae | *Donu* (*Plectropomus* spp.) | 1 |
| Fiji | Serranidae | *Tokula* (*Epinephelus* spp.) | 1 |
| Fiji | Siganidae | *Nuqa* (*Siganus* spp.) | 5 |
| Fiji | Sphyraenidae | *Ogo* (Sphyraenidae) | 7 |
| Fiji | Sphyraenidae | *Silasila* (Sphyraenidae) | 2 |
| Fiji | Syngnathidae | *Ki* (*Corythoichthys* spp.) | 1 |
| Fiji | Syngnathidae | *Ose* (*Corythoichthys* spp.) | 6 |
| Fiji | Trichiuridae | *Beleti* (*Trichiurus* spp.) | 3 |
| Fiji | Trichiuridae | *Tauvisi* (*Trichiurus* spp.) | 1 |
| Tonga | Acanthuridae | Black surgeon | 9 |
| Tonga | Acanthuridae | Short-horned unicorn tang | 2 |
| Tonga | Acanthuridae | Unicornfish | 1 |
| Tonga | Acanthuridae | Unicorn Tang | 5 |
| Tonga | Carangidae | Jacks | 1 |
| Tonga | Coryphaenidae | Mahimahi | 1 |
| Tonga | Gerreidae | *Matu* (*Gerres oyena*) | 3 |
| Tonga | Gerreidae | Silver Biddy | 1 |
| Tonga | Kyphosidae | Rudder fish | 1 |
| Tonga | Labridae | Red-tailed wrasse | 1 |
| Tonga | Leiognathidae | Pony fish | 1 |
| Tonga | Leiognathidae | *Sipesipa* (*Leiognathus equulus*) | 4 |
| Tonga | Lethrinidae | Emperor | 5 |
| Tonga | Lutjanidae | Mutton fish | 9 |
| Tonga | Lutjanidae | Snapper | 3 |
| Tonga | Mugilidae | Grey mullet | 1 |
| Tonga | Mugilidae | Medium-size mullet | 4 |
| Tonga | Mugilidae | Mullet | 1 |
| Tonga | Mullidae | Black-banded goatfish | 3 |
| Tonga | Mullidae | Goatfish | 3 |
| Tonga | Muraenidae | Moray eel | 1 |
| Tonga | Scaridae | Parrotfish | 14 |
| Tonga | Scombridae | Bigeye tuna | 2 |
| Tonga | Scombridae | *Thunnus albacares* | 1 |
| Tonga | Scombridae | Tuna | 2 |
| Tonga | Scombridae | Wahoo Tuna | 1 |
| Tonga | Scombridae | Yellowfin tuna | 5 |
| Tonga | Serranidae | Rock cod | 2 |
| Tonga | Siganidae | Rabbitfish | 5 |
| Tonga | Siganidae | Streamlined spinefoot | 6 |
| Tonga | Sphyraenidae | Great barracuda | 1 |
| Tonga | Stromateidae | Butterfish | 1 |
| Tonga | Unknown | Shark | 1 |
| Tonga | Xiphiidae | Swordfish | 1 |
| Tuvalu | Acanthuridae | *Poneuli (Acanthurus nigricauda)* | 1 |
| Tuvalu | Acanthuridae | *Maono (Acanthurus guttatus)* | 1 |
| Tuvalu | Acanthuridae | *Ponelolo (Acanthurus lineatus)* | 13 |
| Tuvalu | Acanthuridae | *Acanthurus punctatus* | 2 |
| Tuvalu | Acanthuridae | *Manini (Acanthurus triostegus)* | 16 |
| Tuvalu | Acanthuridae | *Kapalagi (Acanthurus xanthopterus)* | 1 |
| Tuvalu | Acanthuridae | *Pokapoka niu (Naso vlamingii)* | 1 |
| Tuvalu | Acanthuridae | *Maninilakau (Naso lituratus)* | 7 |
| Tuvalu | Acanthuridae | *Ume (Naso unicornis)* | 7 |
| Tuvalu | Balistidae | *Melichthys vidua* | 1 |
| Tuvalu | Belonidae | *Platybelone argalus* | 2 |
| Tuvalu | Belonidae | *Tylosurus crocodilus* | 1 |
| Tuvalu | Carangidae | *Alectis ciliaris* | 1 |
| Tuvalu | Carangidae | Carangidae | 1 |
| Tuvalu | Carangidae | *Ferdauia ferdau* | 4 |
| Tuvalu | Carangidae | *Tino Ulua (Caranx ignobilis)* | 5 |
| Tuvalu | Carangidae | *Aseu (Caranx melampygus)* | 9 |
| Tuvalu | Carangidae | *Atule loa (Decapterus macarellus)* | 2 |
| Tuvalu | Carangidae | *Lupolupo (Gnathanodon speciosus)* | 4 |
| Tuvalu | Carcharinidae | *Carcharhinus melanopterus* | 1 |
| Tuvalu | Cirrhitidae | *Paracirrhites arcatus* | 1 |
| Tuvalu | Coryphaenidae | *Coryphaena hippurus* | 1 |
| Tuvalu | Exocoetidae | *Cheilopogon* spp. | 1 |
| Tuvalu | Exocoetidae | *Xocoetus* spp. | 1 |
| Tuvalu | Gempylidae | *Ruvettus pretiosus* | 1 |
| Tuvalu | Gerreidae | *Gerres oyena* | 3 |
| Tuvalu | Holocentridae | *Myripristics murdjan* | 1 |
| Tuvalu | Holocentridae | *Myripristis adusta* | 1 |
| Tuvalu | Holocentridae | *Myripristis* spp. | 1 |
| Tuvalu | Holocentridae | *Sargocentron* spp. | 1 |
| Tuvalu | Holocentridae | *Sargocentron spiniferum* | 4 |
| Tuvalu | Holocentridae | *Sargocentron violaceum* | 2 |
| Tuvalu | Kyphosidae | *Kyphosus vaigiensis* | 1 |
| Tuvalu | Labridae | *Cheilinus undulatus* | 1 |
| Tuvalu | Lethrinidae | *Gnathodentex aurolineatus* | 1 |
| Tuvalu | Lethrinidae | *Gymnocranius grandoculis* | 1 |
| Tuvalu | Lethrinidae | Lethrinidae | 5 |
| Tuvalu | Lethrinidae | *Lethrinidae variegatus* | 2 |
| Tuvalu | Lethrinidae | *Lethrinus harak* | 3 |
| Tuvalu | Lethrinidae | *Lethrinus miniatus* | 4 |
| Tuvalu | Lethrinidae | *Lethrinus obsoletus* | 1 |
| Tuvalu | Lethrinidae | *Lethrinus olivaceus* | 3 |
| Tuvalu | Lethrinidae | *Lethrinus* spp. | 2 |
| Tuvalu | Lethrinidae | *Monotaxis grandoculis* | 4 |
| Tuvalu | Lutjanidae | *Aphareus furca* | 1 |
| Tuvalu | Lutjanidae | *Aprion virescens* | 1 |
| Tuvalu | Lutjanidae | *Gnathodentex aurolineatus* | 3 |
| Tuvalu | Lutjanidae | *Lutjanus kasmira* | 4 |
| Tuvalu | Lutjanidae | Lutjanidae | 1 |
| Tuvalu | Lutjanidae | *Lutjanus bohar* | 1 |
| Tuvalu | Lutjanidae | *Lutjanus fulvus* | 2 |
| Tuvalu | Lutjanidae | *Lutjanus gibbus* | 16 |
| Tuvalu | Lutjanidae | *Lutjanus kasmira* | 1 |
| Tuvalu | Lutjanidae | *Lutjanus monostigma* | 1 |
| Tuvalu | Lutjanidae | *Lutjanus olivaceus* | 1 |
| Tuvalu | Lutjanidae | *Lutjanus semicinctus* | 4 |
| Tuvalu | Lutjanidae | *Pristipomoides multidens* | 1 |
| Tuvalu | Mugilidae | *Crenimugil crenilabis* | 5 |
| Tuvalu | Mugilidae | *Ellochelon vaigiensis* | 2 |
| Tuvalu | Mugilidae | Mugilidae | 1 |
| Tuvalu | Mullidae | *Mulloidichthys flavolineatus* | 2 |
| Tuvalu | Mullidae | *Mulloidichthys vanicolensis* | 3 |
| Tuvalu | Mullidae | *Parupeneus multifasciatus* | 4 |
| Tuvalu | Mullidae | *Parupeneus fuscus* | 1 |
| Tuvalu | Mullidae | *Parupeneus barberinus* | 2 |
| Tuvalu | Pempheridae | *Pempheris oualensis* | 1 |
| Tuvalu | Pomacentridae | *Abudefduf vaigiensis* | 4 |
| Tuvalu | Priacanthidae | *Priacanthus hamrur* | 1 |
| Tuvalu | Scaridae | *Chlorurus microrhinos* | 1 |
| Tuvalu | Scaridae | *Hipposcarus longiceps* | 5 |
| Tuvalu | Scaridae | *Scarus ghobban* | 2 |
| Tuvalu | Scombridae | *Euthynnus affinis* | 4 |
| Tuvalu | Scombridae | *Katsuwonus pelamis* | 6 |
| Tuvalu | Scombridae | *Scomberomorus commerson* | 1 |
| Tuvalu | Scombridae | *Thunnus albacares* | 2 |
| Tuvalu | Serranidae | *Anyperodon leucogrammicus* | 1 |
| Tuvalu | Serranidae | *Cephalopholis argus* | 2 |
| Tuvalu | Serranidae | *Cephalopholis miniata* | 2 |
| Tuvalu | Serranidae | *Cephalopholis* spp. | 3 |
| Tuvalu | Serranidae | *Epinephelus hexagonatus* | 2 |
| Tuvalu | Serranidae | *Epinephelus merra* | 12 |
| Tuvalu | Serranidae | *Epinephelus polyphekadion* | 4 |
| Tuvalu | Serranidae | *Epinephelus fuscoguttatus* | 2 |
| Tuvalu | Serranidae | *Epinephelus polyphekadion* | 1 |
| Tuvalu | Serranidae | *Epinephelus* spp. | 2 |
| Tuvalu | Serranidae | *Plectropomus laevis* | 2 |
| Tuvalu | Siganidae | *Siganus argenteus* | 4 |
| Tuvalu | Siganidae | *Siganus punctatus* | 6 |
| Tuvalu | Sphyraenidae | *Sphyraena barracuda* | 1 |
| Tuvalu | Unknown | shark | 2 |
| Vanuatu | Acanthuridae | Black surgeon | 3 |
| Vanuatu | Acanthuridae | Blackspine surgeon | 1 |
| Vanuatu | Acanthuridae | Convict tang | 2 |
| Vanuatu | Acanthuridae | Humpnose unicornfish | 2 |
| Vanuatu | Acanthuridae | Lined surgeonfish | 7 |
| Vanuatu | Acanthuridae | Orange spine unicornfish | 1 |
| Vanuatu | Acanthuridae | Short-horned unicorn tang | 3 |
| Vanuatu | Acanthuridae | Sleek unicornfish | 1 |
| Vanuatu | Acanthuridae | Streamlined unicorn tang | 3 |
| Vanuatu | Acanthuridae | Striped surgeonfish | 2 |
| Vanuatu | Acanthuridae | White-freckled surgeonfish | 3 |
| Vanuatu | Balistidae | Black patch trigger | 1 |
| Vanuatu | Balistidae | Picasso triggerfish | 5 |
| Vanuatu | Balistidae | Titan triggerfish | 4 |
| Vanuatu | Balistidae | Triggerfish | 1 |
| Vanuatu | Balistidae | Wedge tail triggerfish | 3 |
| Vanuatu | Belonidae | Needlefish | 5 |
| Vanuatu | Caesionidae | Blue & yellow fusilier | 1 |
| Vanuatu | Carangidae | Amberjack | 2 |
| Vanuatu | Carangidae | Bigeye scad | 1 |
| Vanuatu | Carangidae | Bluefin trevally | 4 |
| Vanuatu | Carangidae | Brassy trevally | 1 |
| Vanuatu | Carangidae | Crevalle jacks | 1 |
| Vanuatu | Carangidae | Greater amberjack | 1 |
| Vanuatu | Carangidae | Highfin amberjack | 1 |
| Vanuatu | Carangidae | Rainbow runner | 1 |
| Vanuatu | Carangidae | Snub nose pompano | 1 |
| Vanuatu | Clupeidae | Sardines | 3 |
| Vanuatu | Coryphaenidae | Mahimahi | 1 |
| Vanuatu | Diodontidae | Balloonfish | 1 |
| Vanuatu | Diodontidae | Porcupinefish | 1 |
| Vanuatu | Gerreidae | Black tip silver biddy | 1 |
| Vanuatu | Gerreidae | Longtail silver biddy | 1 |
| Vanuatu | Gerreidae | Oblong silver biddy | 1 |
| Vanuatu | Haemulidae | Diagonal-banded sweetlips | 1 |
| Vanuatu | Haemulidae | Many-spotted sweetlips | 1 |
| Vanuatu | Haemulidae | Oblique-banded sweetlips | 1 |
| Vanuatu | Hemiramphidae | *Hermiramphus far* (spotted halfbeak) | 4 |
| Vanuatu | Hemiramphidae | Spotted half beak | 2 |
| Vanuatu | Holocentridae | Bikini squirrelfish | 1 |
| Vanuatu | Holocentridae | Brick soldierfish | 1 |
| Vanuatu | Holocentridae | Violet squirrelfish | 1 |
| Vanuatu | Holocentridae | White tip soldierfish | 1 |
| Vanuatu | Istiophoridae | Blue marlin Tuna | 1 |
| Vanuatu | Kyphosidae | Top sail drummer | 1 |
| Vanuatu | Labridae | Cigar wrasse | 1 |
| Vanuatu | Labridae | Red-tailed wrasse | 2 |
| Vanuatu | Labridae | Rock mover | 3 |
| Vanuatu | Labridae | Trippletail wrasse | 1 |
| Vanuatu | Labridae | Yellowtail corris | 1 |
| Vanuatu | Lethrinidae | Emperor | 3 |
| Vanuatu | Lethrinidae | Longface emperor | 1 |
| Vanuatu | Lethrinidae | Orange-striped emperor | 8 |
| Vanuatu | Lethrinidae | Thumbprint emperor | 22 |
| Vanuatu | Lutjanidae | Blacktail snapper | 1 |
| Vanuatu | Lutjanidae | Deep sea long tail red snapper (poulet) | 4 |
| Vanuatu | Lutjanidae | Humpback red snapper | 9 |
| Vanuatu | Lutjanidae | Mangrove red snapper | 1 |
| Vanuatu | Lutjanidae | Mutton fish | 6 |
| Vanuatu | Lutjanidae | Ornate jobfish | 1 |
| Vanuatu | Lutjanidae | Snapper | 2 |
| Vanuatu | Lutjanidae | Blacktail snapper | 1 |
| Vanuatu | Lutjanidae | Yellowtail snapper | 1 |
| Vanuatu | Mugilidae | Bluespot mullet | 3 |
| Vanuatu | Mugilidae | Flathead grey mullet | 3 |
| Vanuatu | Mugilidae | Medium sized mullet | 2 |
| Vanuatu | Mugilidae | Mullet | 1 |
| Vanuatu | Mugilidae | Squaretail mullet | 2 |
| Vanuatu | Mullidae | Black-banded goatfish | 2 |
| Vanuatu | Mullidae | Dash-dot goatfish | 5 |
| Vanuatu | Mullidae | Double bar goatfish | 1 |
| Vanuatu | Mullidae | Indian goatfish | 8 |
| Vanuatu | Mullidae | Manybar goatfish | 1 |
| Vanuatu | Mullidae | Sidespot goatfish | 1 |
| Vanuatu | Mullidae | Yellowstriped goatfish | 4 |
| Vanuatu | Nemipteridae | Bream | 1 |
| Vanuatu | Scaridae | Bleekers parrotfish | 3 |
| Vanuatu | Scaridae | Blue barred parrotfish | 1 |
| Vanuatu | Scaridae | Carolines parrotfish | 1 |
| Vanuatu | Scaridae | Daisy parrotfish | 1 |
| Vanuatu | Scaridae | Dusky parrotfish | 1 |
| Vanuatu | Scaridae | Filament-finned parrotfish | 2 |
| Vanuatu | Scaridae | Greenhead parrotfish | 1 |
| Vanuatu | Scaridae | Marbled parrotfish | 1 |
| Vanuatu | Scaridae | Pacific long nose parrotfish | 1 |
| Vanuatu | Scaridae | Pacific slopehead parrotfish | 3 |
| Vanuatu | Scaridae | Palecheek parrotfish | 2 |
| Vanuatu | Scaridae | Parrotfish | 6 |
| Vanuatu | Scaridae | Quoys parrotfish | 1 |
| Vanuatu | Scaridae | Spotted parrot fish | 1 |
| Vanuatu | Scaridae | Steephead parrotfish | 5 |
| Vanuatu | Scombridae | Bluefin trevally | 1 |
| Vanuatu | Scombridae | Dogtooth tuna | 2 |
| Vanuatu | Scombridae | Skipjack Tuna | 2 |
| Vanuatu | Scombridae | Wahoo Tuna | 1 |
| Vanuatu | Scombridae | Yellowfin tuna | 2 |
| Vanuatu | Serranidae | Flagtail grouper | 3 |
| Vanuatu | Serranidae | Honeycomb grouper | 3 |
| Vanuatu | Serranidae | Peacock grouper | 3 |
| Vanuatu | Serranidae | Rock cod | 4 |
| Vanuatu | Serranidae | Strawberry grouper | 1 |
| Vanuatu | Serranidae | White blotched grouper | 1 |
| Vanuatu | Serranidae | Yellow-edged lyretail | 3 |
| Vanuatu | Siganidae | Barred spinefoot rabbitfish | 2 |
| Vanuatu | Siganidae | Dusky rabbitfish | 1 |
| Vanuatu | Siganidae | Gold spotted spinefoot | 1 |
| Vanuatu | Siganidae | Gold-lined spinefoot rabbitfish | 3 |
| Vanuatu | Siganidae | Little spinefoot rabbitfish | 4 |
| Vanuatu | Siganidae | Pencil-streaked rabbitfish | 1 |
| Vanuatu | Siganidae | Rabbitfish | 4 |
| Vanuatu | Siganidae | Seagrass rabbitfish | 2 |
| Vanuatu | Siganidae | Streamlined spinefoot | 3 |
| Vanuatu | Siganidae | Vermiculated spinefoot rabbitfish | 7 |
| Vanuatu | Siganidae | White-spotted spinefoot | 10 |
| Vanuatu | Sphyraenidae | Barracuda | 1 |
| Vanuatu | Sphyraenidae | Great barracuda | 2 |

**Supplementary Table 4:** Table showing the number of individuals sampled for microplastics for each family (MP Replication), the mean (± SE) of microplastic concentration for each family (MP Concentration), and the total number of times the family was mentioned as being caught within the fishing grounds during interviews (Catch Mentions), presented by country.

| **Country** | **Family** | **MP Replication** | **MP Concentration** | **Catch Mentions** |
| --- | --- | --- | --- | --- |
| **Fiji** | Scombridae | 3 | 5.67 ± 1.2 | 14 |
|  | Serranidae | 4 | 3.00 ± 2.12 | 27 |
|  | Lethrinidae | 86 | 2.35 ± 0.24 | 38 |
|  | Lutjanidae | 7 | 3.00 ± 1.40 | 23 |
|  | Scaridae | 11 | 1.82 ± 0.40 | 11 |
|  | Siganidae | 10 | 2.10 ± 0.43 | 5 |
|  | Hemiramphidae | 20 | 1.45 ± 0.45 | 7 |
|  | Mullidae | 14 | 2.07 ± 0.43 | 2 |
|  | Sphyraenidae | 20 | 1.00 ± 0.31 | 9 |
|  |  |  |  |  |
| **Tonga** | Siganidae | 8 | 0.75 ± 0.31 | 11 |
|  | Scaridae | 20 | 0.50 ± 0.69 | 14 |
|  | Acanthuridae | 4 | 0.25 ± 0.50 | 17 |
|  | Mugilidae | 4 | 0.75 ± 0.50 | 6 |
|  | Lethrinidae | 59 | 0.93 ± 0.15 | 5 |
|  | Mullidae | 20 | 0.55 ± 0.20 | 6 |
|  | Serranidae | 5 | 0.40 ± 0.24 | 2 |
|  | Lutjanidae | 5 | 0.00 ± n/a | 12 |
|  | Carangidae | 5 | 3.40 ± 1.29 | 1 |
|  | Labridae | 4 | 0.50 ± 0.50 | 1 |
|  |  |  |  |  |
| **Tuvalu** | Acanthuridae | 33 | 1.00 ± 0.31 | 49 |
|  | Lutjanidae | 8 | 1.00 ± 0.63 | 37 |
|  | Mullidae | 21 | 1.23 ± 0.39 | 12 |
|  | Siganidae | 4 | 1.25 ± 0.89 | 10 |
|  | Holocentridae | 13 | 1.15 ± 0.49 | 10 |
|  | Lethrinidae | 64 | 0.58 ± 0.22 | 26 |
|  | Scaridae | 7 | 0.71 ± 0.67 | 8 |
|  | Serranidae | 39 | 0.28 ± 0.28 | 36 |
|  | Sphyraenidae | 4 | 0.50 ± 0.89 | 1 |
|  |  |  |  |  |
| **Vanuatu** | Mullidae | 15 | 0.13 ± 0.09 | 22 |
|  | Lethrinidae | 34 | 0.06 ± 0.06 | 34 |
|  | Scaridae | 46 | 0.07 ± 0.05 | 30 |
|  | Serranidae | 14 | 0.07 ± 0.07 | 18 |
|  | Hemiramphidae | 34 | 0.21 ± 0.07 | 6 |
|  | Haemulidae | 3 | 0.32 ± 0.33 | 3 |
|  | Carangidae | 73 | 0.04 ± 0.02 | 14 |
|  | Mugilidae | 22 | 0.0 ± n/a | 11 |
|  | Labridae | 14 | 0.0 ± n/a | 8 |
|  | Belonidae | 4 | 0.0 ± n/a | 5 |
|  | Siganidae | 17 | 0.0 ± n/a | 38 |
|  | Acanthuridae | 12 | 0.0 ± n/a | 28 |
|  | Lutjanidae | 35 | 0.0 ± n/a | 26 |
|  | Kyphosidae | 5 | 0.0 ± n/a | 1 |

**Supplementary Table 5:** Table showing the number of individuals sampled for microplastics for each family (MP Replication), the mean (± SE) of microplastic concentration for each family (MP Concentration), and the total number of times the family was mentioned as being caught within the fishing grounds during interviews (Catch Mentions), presented by country.

| **Family** | **MP Replication** | **MP Concentration** | **Catch Mentions** |
| --- | --- | --- | --- |
| Scombridae | 3 | 5.67 ± 1.20 | 46 |
| Lethrinidae | 243 | 1.22 ± 0.13 | 103 |
| Acanthuridae | 49 | 0.69 ± 0.30 | 106 |
| Siganidae | 39 | 0.82 ± 0.33 | 64 |
| Lutjanidae | 55 | 0.53 ± 0.28 | 98 |
| Mullidae | 70 | 0.97 ± 0.25 | 42 |
| Serranidae | 62 | 0.42 ± 0.26 | 83 |
| Scaridae | 84 | 0.45 ± 0.23 | 63 |
| Carangidae | 79 | 0.30 ± 0.23 | 67 |
| Sphyraenidae | 26 | 0.85 ± 0.49 | 14 |
| Holocentridae | 18 | 0.83 ± 0.49 | 14 |
| Hemiramphidae | 54 | 0.67 ± 0.28 | 13 |
| Kyphosidae | 7 | 1.14 ± 0.79 | 6 |
| Mugilidae | 26 | 0.12 ± 0.41 | 35 |
| Haemulidae | 3 | 0.33 ± 1.20 | 6 |
| Labridae | 18 | 0.11 ± 0.49 | 12 |
| Caesionidae | 8 | 0.38 ± 0.74 | 1 |
| Belonidae | 4 | 0.00 ± n/a | 5 |

**Supplementary Figure 1**. Exposure Index workflow: Catch Score * MP Score = Exposure Index (higher values = higher risk).


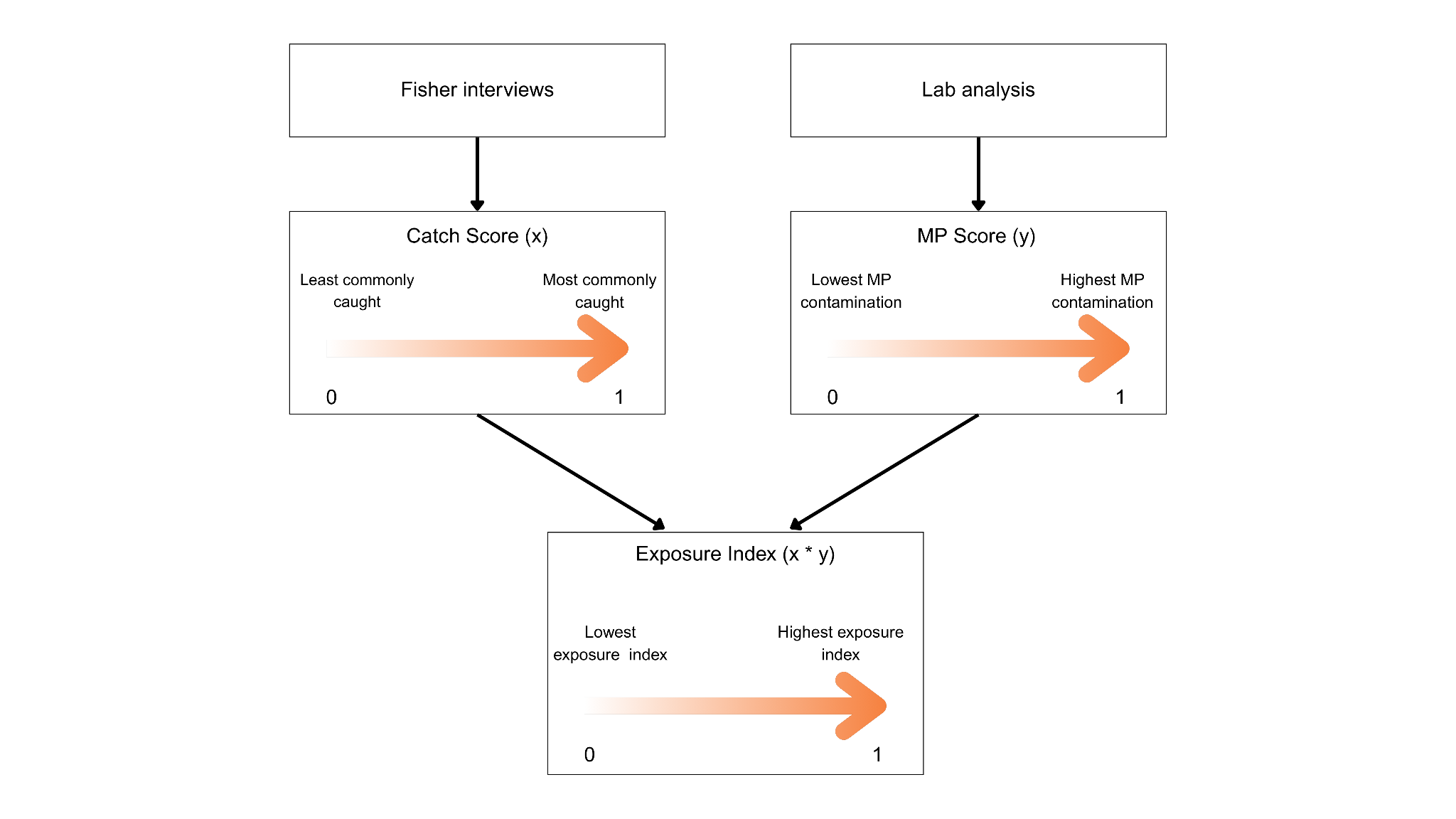


|  |  |  |  |  |  |  |
| --- | --- | --- | --- | --- | --- | --- |
|  |  |  |  |  |  |  |
|  |  |  |  |  |  |  |
|  |  |  |  |  |  |  |
|  |  |  |  |  |  |  |
|  | |  |  |  |  |  |

**Supplementary Figure 2:** Principal component analysis (PCA) biplot of fish family composition across countries, based on the percentage contribution of the ten most abundant families to total recorded catch. Points represent countries, while arrows indicate the contribution of each fish family to the principal components. Family labels were adjusted using repelling algorithms to reduce overlap. The analysis highlights patterns in community composition among countries based on dominant fish families.

**
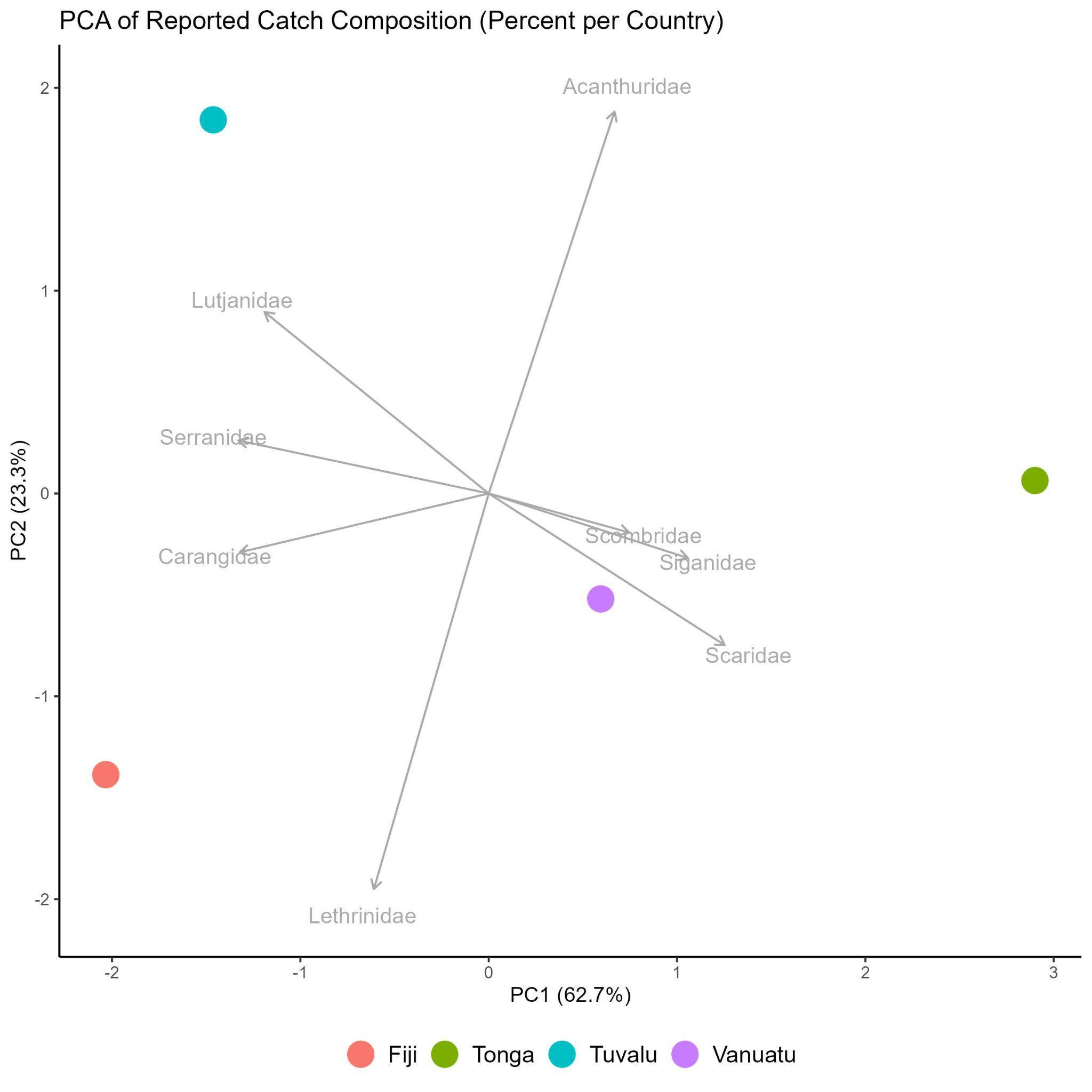
**

**Supplementary Figure 3:** Gender disaggregated data on families reported as common catch (1.0 = 100% reported by females, 0 = reported consistently among genders; -1.0 = 100% reported by males), including all families reported (i.e. including those excluded without microplastics data that were excluded from the exposure risk calculations).


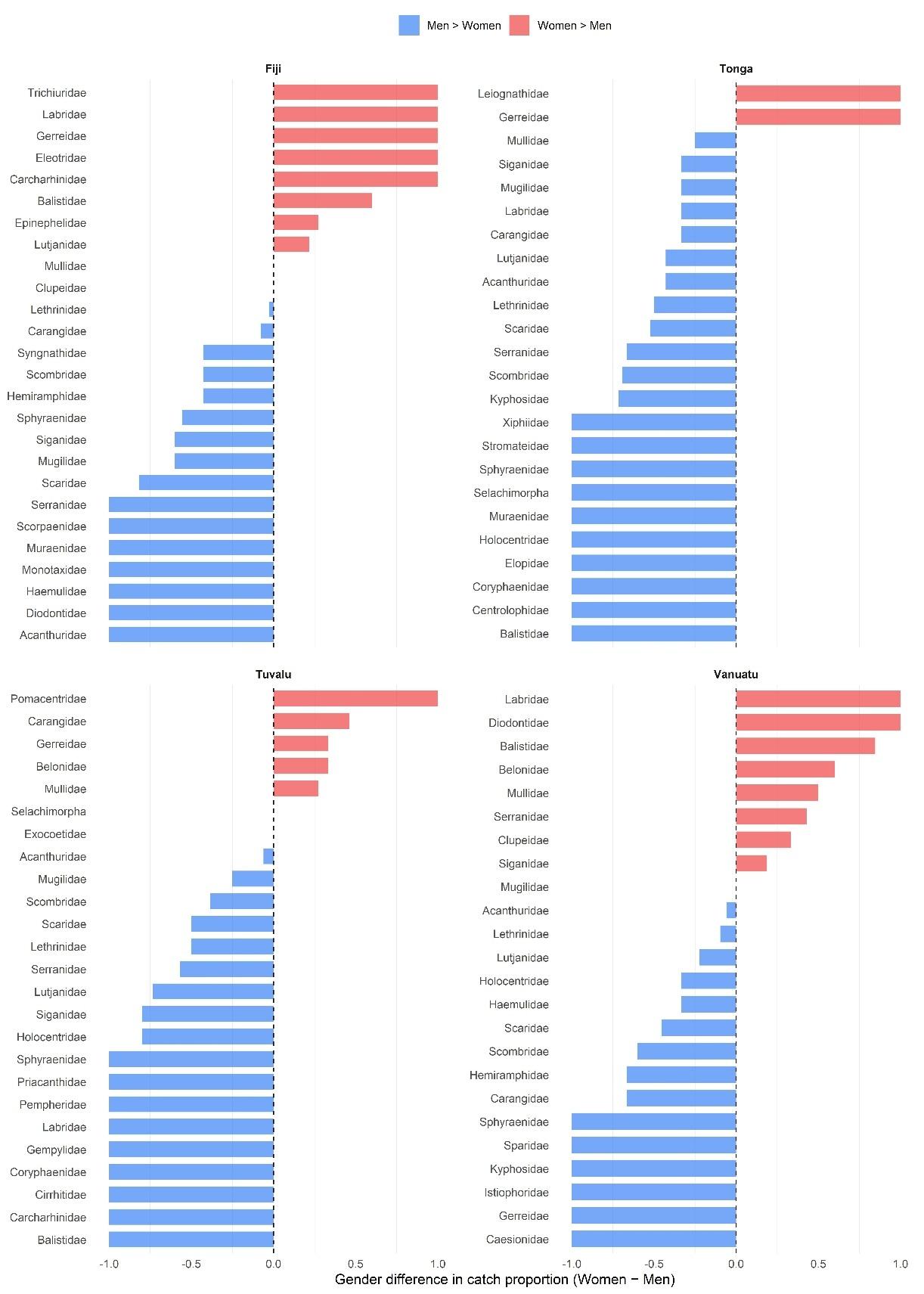

Supplement: Ford et al. supplementary material [file S2754720526100274sup001.docx]
